# Effect of thioredoxin on the immunogenicity of the recombinant P32 protein of lumpy skin disease virus

**DOI:** 10.14202/vetworld.2022.2384-2390

**Published:** 2022-10-11

**Authors:** Kanat Tursunov, Laura Tokhtarova, Darkhan Kanayev, Raikhan Mustafina, Kanatbek Mukantayev

**Affiliations:** 1 Laboratory of Immunochemistry and Immunobiotechnology, National Center for Biotechnology, 010000, Nur-Sultan, Kazakhstan; 2 Department of Veterinary Sanitation, Faculty of Veterinary and Animal Husbandry Technology, S. Seifullin Kazakh Agro Technical University, 010011, Nur-Sultan, Kazakhstan

**Keywords:** immunogenicity, lumpy skin disease virus, P32 protein, recombinant antigen, thioredoxin

## Abstract

**Background and Aim:**

The rapid spread of lumpy skin disease (LSD) globally poses a serious threat to the agricultural sector. The timely and accurate diagnosis of the disease is crucial to control LSD. This study aimed to determine the effect of thioredoxin on the immunogenicity of the recombinant P32 (rP32) protein of LSD virus (LSDV). Since the P32 protein is poorly soluble, it is often expressed by adding an auxiliary sequence of a highly soluble partner protein such as thioredoxin.

**Materials and Methods:**

The P32 gene fragment was amplified using a polymerase chain reaction from genomic DNA used as a template. The resulting DNA fragments were cloned into the pET32a vector, and transformed into Escherichia coli BL21 (DE3) cells through electroporation. Purification of the rP32 protein was performed using a HisTrap column. Purified rP32 protein fused with thioredoxin (rP32Trx) was characterized by western blotting, liquid chromatography with tandem mass spectrometry and indirect enzyme-linked immunosorbent assay (ELISA).

**Results:**

Indirect ELISA revealed that, despite the lower molecular weight, the main part of the antibodies in the serum of immunized mice was directed against thioredoxin and not the target P32 protein. Thus, the antibody titers against rP32Trx were 1:102400, whereas antibody titers against heterologous recombinant 3BTrx and PD1Trx proteins were 1:25600 and 1:51200, respectively. Concurrently, the antibodies did not bind to the heterologous recombinant PD1 protein, which did not contain thioredoxin.

**Conclusion:**

The results showed that the rP32 protein fused with the partner protein thioredoxin could not be used to obtain polyclonal and monoclonal antibodies. However, the recombinant fusion protein rP32Trx can be used to develop a serological test to detect antibodies, since antibodies against thioredoxin were not detected in the animal sera.

## Introduction

Lumpy skin disease (LSD) is a highly contagious disease affecting farm animals, causing significant economic damage to the livestock industry. The disease is caused by the LSD virus (LSDV) belonging to the genus *Capripoxvirus* of the *Poxviridae* family. The disease presents with fever, depression, decreased productivity, and characteristic skin nodules [1, 2]. LSD is currently widespread in the Middle East, the Balkans, Europe, the Russian Federation, China, Kazakhstan, and Thailand [3–7]. Following the recommendations of the World Organization for Animal Health (OIE), LSD is diagnosed using serological methods, namely agar gel immunodiffusion reaction, indirect immunofluorescent antibody test, enzyme-linked immunosorbent assay (ELISA), and western blotting [8]. Comparative analyses of the recommended diagnostic tests have demonstrated the high efficiency, sensitivity, and specificity of ELISA while analyzing multiple samples [9, 10]. The diagnostic sensitivity and specificity of ELISA are 96% and 95%, respectively [11]. ELISA based on the P32 protein of LSDV allows rapid and reliable testing to detect virus-specific antibodies in animals [12]. A protocol has been developed to produce truncated recombinant P32 (rP32) proteins in *Escherichia* *coli* cells. In addition, western blotting and ELISA using the rP32 protein of LSDV demonstrated diagnostic potential for serological surveillance of infection in sheep and goats [13].

Various cell systems using bacterial, mammalian, or insect cells are employed to produce recombinant proteins [14]. *Escherichia coli* is most often used to obtain recombinant antigens due to its rapid growth and inexpensive nutrient media. However, despite their clear advantages, *E. coli* cells also have some disadvantages. Heterologous proteins are often expressed as insoluble forms in inclusion bodies. This can affect the biological activity of the protein and can hinder the prospects of its use in developing diagnostic systems. One of these proteins is the rP32 protein of the LSDV [13, 15]. However, difficulties associated with the purification process and the low level of the rP32 protein expression (due to poor solubility) are significant challenges for developing a practical diagnostic test. There are several methods for increasing the solubility of recombinant proteins expressed in *E. coli*. Fusion of the recombinant protein with GroES, MalE protein, thioredoxin, or glutathione S-transferase resulted in high productivity. In addition, thioredoxin makes it possible to obtain proteins in the cytoplasm and other compartments of *E. coli* [16, 17]. Thioredoxin is a 12 kDa protein found in bacteria, yeast, plants, and animals [18]. An essential property of thioredoxin is its ability to increase the solubility of hybrid proteins [19]. The advantage of thioredoxin as a partner protein lies in its highly efficient translation in bacterial cells; it accumulates in large amounts in a soluble form (up to 40% of the total cellular protein) [20]. However, the efficiency of increasing the solubility of the hybrid protein does not depend on the addition of thioredoxin to the N- or C-terminus [21].

Whether thioredoxin affects the immunological properties of recombinant proteins used to obtain monoclonal antibodies remains unclear. This study aimed to determine the immunological properties of the rP32 antigen from LSDV when fused with thioredoxin (rP32Trx) to obtain monoclonal antibodies.

## Materials and Methods

### Ethical approval

This study was approved by the Ethical Committee of the National Center for Biotechnology, Nur-Sultan, Republic of Kazakhstan (IRB00013497).

### Study period and location

The study was conducted from September 2021 to April 2022 in the Laboratory of Immunochemistry and Immunobiotechnology, National Center for Biotechnology, Nur-Sultan, Kazakhstan.

### Bacterial strains, plasmids, and culture media

The laboratory strains *E. coli* DH5α and *E. coli* BL21 (DE3) (Sigma-Aldrich, Darmstadt, Germany) were used in this study. The pGEM-TEasy plasmid (Promega, Madison, WI, USA) and pET32a expression plasmid (Sigma-Aldrich) were used for cloning. The microbial strains were cultured in Luria–Bertani (LB) broth and agar medium.

### DNA and primer design

Primers were designed using Lasergene 6 software (https://www.dnastar.com/software/lasergene/) based on the P32 LSDV gene sequence obtained from the NCBI GenBank database (https://www.ncbi.nlm.nih.gov/genome), and synthesized at the National Center for Biotechnology, Republic of Kazakhstan (Table-1). Genomic DNA was obtained from the Laboratory of Applied Genetics “National Center for Biotechnology,” Republic of Kazakhstan.

**Table-1:** Primers for amplification of gene P32 of LSDV.

Target	Primer	Sequence (5′–3′)	Restriction enzyme	Length (bp)
P32 LSDV	P32 fw	GTTGGTCGCGAAATTTCAGATGTA	-	24
	P32 rev	GTAAGAAAAATCAGGAAATCTATG	-	24
	P32fsr	*GAATTCCCATGG*TGGTTGGTCGCGAAATT	EcoRI, NcoI	29
	P32rsr	*AAGCTTC*TCGAGGTAAGAAAAATC	XhoI	24

LSDV=Lumpy skin disease virus

### Laboratory animals

The organization had all the necessary conditions for maintaining laboratory mice according to the requirements of their physiology. White laboratory mice (n = 12) of age 10–12 weeks, weighing 22–24 g, were used in this study. Animals were provided ad libitum access to standard chow and water.

### Amplification of *P32* gene fragment

Genomic DNA was used as a template for the amplification of the *P32* gene fragment using polymerase chain reaction (PCR). For the first stage, the reaction was carried out using 2.5 μL of 10× Taq buffer (Thermo Fisher Scientific, Vilnius, Lithuania), 1 μL of genomic DNA, 1 μL (10 pmol/μL) of primers, 2.5 μL of dNTP Mix (2 mM each), 2.5 μL 25 mM MgCl, and 0.5 μL of Taq DNA polymerase (Thermo Fisher Scientific), brought up to 25 μL using nuclease-free water (Sartorius, Israel). Denaturation was carried out at 95°C for 1 min, followed by 30 cycles at 95°C for 1 min, primer annealing at 58°C for 1 min, and elongation at 72°C for 1 min. For the second stage, the reaction was performed in a 50 μL volume containing 5 μL of 10× Phusion buffer (Thermo Fisher Scientific), 1 μL of the PCR product from the first stage as a template, 1 μL (10 pmol/μL) of forward and reverse primers with restriction sites (NcoI/XhoI), 1 μL of 10 mM dNTP Mix (200 μM each), and 0.5 μL of Phusion High-Fidelity DNA polymerase (Thermo Fisher Scientific). Denaturation was performed at 98°C for 30 s, primer annealing at 60°C for 40 s, and elongation at 72°C for 40 s. The amplified PCR products were analyzed by 1% agarose gel electrophoresis. The DNA band of the predicted size (780 bp) was purified using the Gel Extraction Kit (Invitrogen, Vilnius, and Lithuania) and cloned into the pGEM-TEasy plasmid for selection and sequencing. Three positive clones were selected and sequenced using an ABI PRISM 310 Genetic Analyzer.

### Cloning and expression of recombinant protein

The *P32* nucleotide sequence was excised from the pGEM-TEasy vector using the NcoI and XhoI restriction enzymes for the pET32a plasmid. The resulting DNA fragments were separated by electrophoresis, purified from the gel, and cloned into the pET32a vector using T4 ligase (Thermo Fisher Scientific). The resulting ligation mixture was cloned into E. coli DH5α cells for production, and the vector was isolated from the bacterial culture using a MidiPrep Kit (Invitrogen, Vilnius, Lithuania), following the manufacturer’s instructions. The vector was next transformed into *E. coli* BL21 (DE3) cells through electroporation. The cells were seeded onto LB agar with ampicillin and cultured overnight at 37°C. Several positive colonies were selected and cultured in LB/ampicillin for 16 h at 37°C in an incubator shaker (200 rpm) to determine the expression. The overnight culture was inoculated into 100 mL of LB/ampicillin and incubated at 37°C with shaking at 200 rpm until OD600 was 0.6. Expression of the recombinant protein was determined by adding various concentrations (0.1, 0.2, 0.5, and 0.8 mM) of isopropyl-β-d-1-galactopyranoside (IPTG). Cells were incubated at two different temperatures (37°C and 25°C) to determine the optimal incubation temperature. Aliquots of 5 mL were collected every 2 h, before and after the addition of IPTG, and pelleted by centrifugation at 4000× *g* at 4°C for 10 min.

### Cell lysis and protein purification

The resulting bacterial pellet was resuspended and lysed using an ultrasonic homogenizer. After centrifugation, the pellet was resuspended in a buffer containing 1 M urea, incubated for 30 min at 25°C, and centrifuged at 20,000× *g* for 30 min. The supernatant was collected; the pellet was dissolved in a buffer containing 5 M urea, and sonicated. The purification procedure was repeated using a buffer containing 8 M urea. The recombinant protein was purified using metal affinity chromatography with HisTrap columns (Cytiva, Uppsala, Sweden), following the manufacturer’s instructions. Recombinant protein was eluted using a linear gradient of imidazole buffer (50–500 mM). The resulting fractions were analyzed using 12% sodium dodecyl sulfate-polyacrylamide gel electrophoresis (SDS-PAGE). The samples were mixed with a 3× dilution buffer, boiled in a water bath for 5 min at 95°C, and cooled. Proteins were separated at a voltage of 100 V. The gel was stained for 1 h in a Coomassie R-250 solution and washed with several changes of a decolorizing solution until the background color completely disappeared.

### Western blotting

The presence of the target recombinant protein was determined by western blotting. The protein fractions were separated using SDS-PAGE and transferred to a nitrocellulose membrane, 0.45 μm (GE Healthcare Life Sciences, UK). The membrane was blocked with 1% bovine serum albumin (Abcam, Cambridge, MA, USA) for 1 h at 25°C, and washed 3 times with phosphate-buffered saline containing tween-20 (PBS-T). Next, monoclonal antibodies against His-tag labeled with horseradish peroxidase (HRP) (Qiagen, Hilden, Germany) were added at a dilution of 1:2000 and incubated for 1 h at 25°C, in an orbital shaker. After washing the membrane, 4-chloro-1-naphthol (Sigma-Aldrich, St. Louis, MO, USA) and 0.03% H_2_O_2_ were added. The reaction was stopped by washing the membrane with distilled water.

### Nanoscale liquid chromatography and tandem mass spectrometry (nano LC-MS/MS)

The amino acid sequences of the proteins were determined using mass spectrometry. The isolated and purified rP32Trx protein was prepared for mass spectrometric analysis using standard protein denaturation, disulfide bond reduction, and tryptic hydrolysis. Protein bands were excised from the gel and divided into 1 × 1 mm fragments. Protein digestion was performed by adding trypsin (10 ng/μL) dissolved in 50 mM ammonium bicarbonate, followed by overnight incubation at 37°C. Samples were dried on a SpeedVac vacuum concentrator and purified using Pierce™ C18 Tips and a 10 mL bed (Thermo Fisher Scientific, Rockford, IL, USA). Peptides were separated by HPLC and analyzed using LC-MS/MS on an Acclaim™ PepMap™ (Thermo Scientific, Rockford, IL, USA) RSLC column. The obtained spectra were processed using the MASCOT database (http://www.matrixscience.com/).

### Immunization of mice

Four groups of mice (n = 3 in each group) were used for intraperitoneal immunization. In the first group, each mouse was injected with 25 μg of antigen per mouse. The mice in the second and third groups were injected with 50 μg and 100 μg antigen, respectively. The mice in the fourth group (control) were injected with sterile PBS. The antigen was injected four times at an interval of 10 days. Before immunization and 2 days after the second, third, and fourth injections of the antigen, blood was collected from the tail vein to obtain serum. On the 1^st^ day, the antigen was injected with complete Freund’s adjuvant (Sigma-Aldrich) at a 1:1 ratio. The second immunization was performed with incomplete Freund’s adjuvant (Sigma-Aldrich) at the same ratio. Subsequently, antigen was injected in PBS (pH 7.2).

### Indirect ELISA

The rP32Trx antigen (0.6 mg/mL in carbonate-bicarbonate buffer; pH 9.6) was immobilized onto a 96-well plate. The unbound proteins were removed using PBS-T. Blocking was performed by adding 3% skimmed milk and incubating for 40 min at 37°C. The sera of the mice were added at a dilution of 1:100, followed by a double titration. Next, anti-mouse HRP conjugate (Sigma-Aldrich) was added at a dilution of 1:20,000. Further, o-phenylenediamine dihydrochloride substrate was added to develop color, and the reaction was stopped by adding 0.2 M sulfuric acid. The optical density was measured at a wavelength of 490 nm using a plate spectrophotometer 680 (BioRad, Italy).

## Results

### Amplification, cloning, and expression of the *P32* gene of the LSDV

**Figure-1:**
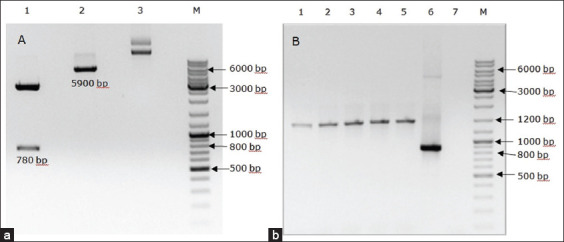
Electrophoresis of restriction-digested and polymerase chain reaction (PCR) products on 1% agarose gel. (a) Lane 1: Restriction analysis of pGEM-T/P32, Lane 2: pET32a plasmid digested with enzymes, Lane 3: pET32a plasmid without enzyme treatment; Lane M: DNA ladder (#SM0333, Thermo Fisher Scientific). (b) Colony PCR. Lanes 1–5: Positive clones, Lane 6: Positive control, Lane 7: Negative control, Lane M: DNA ladder (#SM0333, Thermo Fisher Scientific).

Using PCR and the appropriate primers, a fragment of the *P32* gene of the LSDV of bovine animals (~780 bp) was amplified. The amplified product was cloned into the pGEM-TEasy plasmid (Promega) and transformed into E. coli DH5α. Based on the results of the blue-white selection, positive clones were cultured in a large volume of nutrient medium. After purification, the plasmid pGEM-T/P32 was digested with Nco1 and Xho1 restriction enzymes and cloned into the pET32a expression vector (Figure-1a). The plasmid was transformed into *E. coli* BL21(DE3) cells and incubated on a solid nutrient medium supplemented with ampicillin (prepared by the authors). All clones selected for PCR screening using commercial T7 primers yielded a product of 1200 bp, which was the expected size (Figure-1b).

Positive clones were cultured in LB medium supplemented with various concentrations of IPTG to determine the expression of pET32/P32LSDV plasmids. Samples were collected at 0, 2, 4, 6, and 18 h after induction and analyzed using SDS-PAGE. The IPTG concentration >0.2 mM did not further increase the expression of the rP32Trx protein. In addition, 150 rpm and 6 h incubation at 25°C was sufficient, and further incubation is not recommended since the amount of target protein does not increase notably.

### Purification of rP32Trx protein

**Figure-2:**
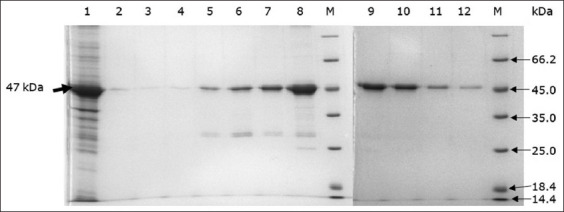
Sodium dodecyl sulfate-polyacrylamide gel electrophoresis of rP32Trx protein purified using metal affinity chromatography. Lane 1: Supernatant of the *Escherichia* coli BL21(DE3)/P32Trx after ultrasonication, Lane 2: Wash buffer, Lanes 3 and 4: 50 mM imidazole eluent, Lanes 5 and 6: 100 mM imidazole eluent, Lanes 7 and 8: 150 mM imidazole eluent, Lanes 9–12: 500 mM imidazole eluent, Lane M: Molecular weight markers (#26610, Thermo Fisher Scientific).

The rP32Trx protein was produced in an insoluble form; therefore, buffers with 1 M, 5 M, and 8 M urea were used for its isolation. The highest protein yield was obtained with 8 M urea buffer (data not shown). The rP32Trx protein was purified on a HisTrap Ni-NTA column. Sulfate-polyacrylamide gel electrophoresis analysis of the purified protein fractions showed that the fractions contained mainly a ~47 kDa protein, corresponding to the predicted rP32Trx protein (Figure-2). The highest protein yield was observed 150 mM imidazole as eluate; however, purer protein preparations were obtained with 500 mM imidazole.

**Figure-3:**
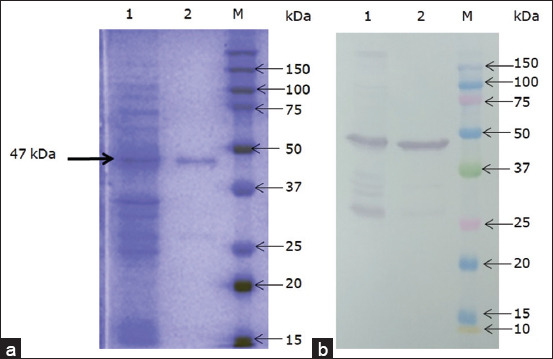
Sodium dodecyl sulfate-polyacrylamide gel electrophoresis (a) and western blotting (b) of rP32Trx protein. Line 1: Total cell lysate of *Escherichia coli* BL21(DE3) containing pET32a/P32, 6 h incubation after isopropyl-β-D-1-galactopyranoside induction; Line 2: purified P32 protein (500 mM imidazole); Lane M: Molecular weight markers (#1610375, BioRad).

Western blotting using anti-His-tag monoclonal antibodies revealed a ~47 kDa protein (Figure-3).

### Nano LC-MS/MS spectrometry

**Figure-4:**
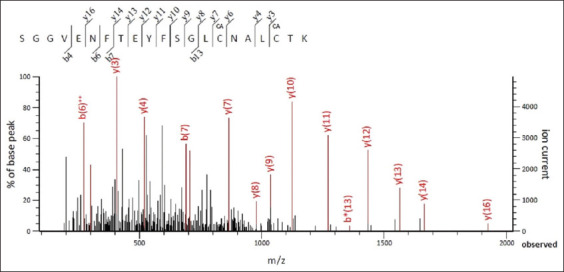
Liquid chromatography and tandem mass spectrometry spectrum of rP32Trx frameshiftprotein.

Analysis of the studied samples was carried out using the MASCOT database, which contains theoretical sets of tryptic peptides for all known amino acid sequences. Figure-4 shows the LC-MS/MS spectrum of the sample after 16 h of tryptic hydrolysis.

The LC-MS/MS spectrum showed that the set of tryptic peptides corresponded to the P32 protein of LSDV. The amino acid sequences correspond to fragments of the nucleotide sequences inserted into the plasmid pET32a for the expression of rP32 in LSDV. This allowed us to conclude that there was no frameshift during the expression of the target protein.

Mice immunization experiments showed that an antigen concentration of 100 μg is toxic to animals. Therefore, mice from this group were not used in further experiments. When white mice were immunized with an antigen at the concentrations of 25 μg and 50 μg, the titers of antisera in indirect ELISA were 1:25600 and 1:102400, respectively (Table-2).

**Table-2:** Immunogenicity of rP32Trx protein in mice.

Serum dilution	rP32Trx antigen concentration, µg/head (OD 490 nm)	

	25	50
1:12800	0.257	0.575
1:25600	0.169*	0.357
1:51200	0.108	0.218
1:102400	0.073	0.157*

* OD values corresponding to antibody titer. Maximum OD of the control well was 0.74

Table-2 shows that the antibody titers in the mice group immunized with 50 μg antigen are significantly higher than at 25 μg. However, how thioredoxin affects antibody production remains unclear on protein fusion immunization. Therefore, to determine the antibody titer directed against thioredoxin and not the target protein, indirect ELISA was performed with other recombinant proteins, fused with thioredoxin (recombinant PD1Trx and non-structural protein of foot-and-mouth disease virus 3BTrx), as well as recombinant PD1 without thioredoxin (Table-3).

**Table-3:** Determination of the cross-reactivity of anti-thioredoxin serum antibodies from immunized mice.

Serum dilution	Recombinant antigens (Serum OD 490 nm)			

	rP32Trx	r3BTrx	rPD1Trx	rPD1
1:12800	1.009	0.303	0.525	0.034
1:25600	0.512	0.182*	0.283	0.045
1:51200	0.305	0.116	0.162*	0.033
1:102400	0.177*	0.090	0.075	0.037

* OD values corresponding to antibody titer

As shown in Table-3, the antibody titers against rP32Trx showed cross-reactivity with heterologous proteins containing thioredoxin. In indirect ELISA, the titer of antibodies in the serum of mice with the r3B/Trx and rPD1Trx antigens was 1:25600 and 1:51200, respectively. Indirect ELISA using rPD1 as an antigen did not reveal any antibodies. These results suggest that the cross-reactivity in the indirect ELISA is related to thioredoxin. When mice were immunized with rP32Trx, a significant amount of antibodies against thioredoxin, rather than the target P32 protein, were found in the sera of mice.

## Discussion

The high contagiousness of LSDV and the intensive spread of the disease in the last decade have prompted researchers to revise the diagnostic paradigm. According to the OIE guidelines, several methods can be used for the serological diagnosis of LSD [8]. ELISA has the advantages of the low cost per reaction, speed, and the ability to analyze multiple samples in one assay [22]. Several ELISA-based tests using monoclonal antibodies and cell culture lysates have been developed to diagnose sheeppox, goatpox, and LSD. However, the use of cell culture substantially increases the cost of the final product because of the use of expensive materials, equipment, specialized laboratories, and trained personnel. In addition, working with the virus requires compliance with special biosafety rules. The use of recombinant proteins is a feasible approach. The production of recombinant proteins in bacterial cells dramatically simplifies this process, making it possible to standardize all stages and reduce production costs. Structural analysis of *Capripoxvirus* proteins [23] revealed the presence of a highly immunogenic P32 protein. The rP32 protein based on the pET32a vector was used to develop an ELISA with high sensitivity and specificity [13]. However, the effect of thioredoxin on antibody production has not been reported to date.

In this study, the rP32 protein was obtained from LSDV fused with thioredoxin. The protein was expressed in a prokaryotic system, which is simple and inexpensive compared with other systems such as *Pichia pastoris* or insect cells [14, 24, 25]. The predicted molecular weight of the fusion protein (~47 kDa) was consistent with the SDS-PAGE results, and literature data [13]. Analysis of the amino acid sequence of the tryptic peptide SGGVENFTEYFSGLCNALCTK by mass spectrometry showed correspondence to the P32 protein of the LSDV (score = 2288), which additionally confirms the expression of the target protein.

The rP32Trx fusion protein is required because of the low expression in *E. coli* due to the formation of an insoluble form of the protein [13]. The use of partner proteins, such as thioredoxin, increased the solubility of recombinant proteins and the yield of the protein from the IPTG-induced culture [20, 21]. However, it is necessary to consider the effect of thioredoxin on the immunological properties of the P32 protein intended to produce monoclonal antibodies. For these purposes, rP32Trx was used to determine immunogenicity in laboratory mice. The sera of immunized mice were studied using indirect ELISA, and the optimal protein concentration, blocking buffer, and dilution of the conjugate were determined. The results showed significant cross-reactivity of the serum antibodies with heterologous proteins fused with thioredoxin. However, the serum containing antibodies against rP32Trx was not positive for antibodies against the heterologous rPD1 protein (without thioredoxin). The data indicated the production of antibodies against thioredoxin in the immunized mice.

## Conclusion

The recombinant fusion rP32Trx protein of LSDV expressed in *E. coli* cells was successfully purified and identified using western blotting and mass spectrometry. It was shown that the use of the P32 protein fused with thioredoxin to obtain both monoclonal and polyclonal antibodies is not advisable. A significant proportion of the antibodies in the sera of immunized mice was directed against thioredoxin and not against the target protein rP32. This greatly complicates the process of obtaining monoclonal antibodies using hybridoma technology. However, theoretically, the rP32Trx protein can be used to develop a serological test for diagnostic purposes since there are no antibodies to thioredoxin in the serum of animals.

## Authors’ Contributions

KT and RM: Conceptualization and design of the study. KT, LT, and DK: Performed the study (gene amplification, cloning, expression and purification of proteins, LC-MS/MS chromatography, and immunization). KT and KM: Prepared and wrote the original draft. KM and RM: Reviewed and edited the manuscript. All authors have read and approved the final manuscript.
